# Hyperlipidemia and Statins Affect Neurological Outcome in Lumbar Spine Injury

**DOI:** 10.3390/ijerph120100402

**Published:** 2015-01-05

**Authors:** Wu-Fu Chung, Shih-Wei Liu, Peng-Yuan Chang, Feng-Shu Lin, Li-Fu Chen, Jau-Ching Wu, Yu-Chun Chen, Laura Liu, Wen-Cheng Huang, Henrich Cheng, Su-Shun Lo

**Affiliations:** 1 Department of Emergency Medicine, National Yang-Ming University Hospital, I-Lan 260, Taiwan; E-Mails: wolfchung2001@yahoo.com.tw (W.-F.C.); shihweiliu123@gmail.com (S.-W.L.); 12206@ymuh.ym.edu.tw (L.-F.C.); 2 Department of Neurosurgery, Neurological Institute, Taipei Veterans General Hospital, Taipei 112, Taiwan; E-Mails: acidbummer@gmail.com (P.-Y.C.); wchuang@vghtpe.gov.tw (W.-C.H.); hc_cheng@vghtpe.gov.tw (H.C.); 3 Department of Pharmacy, National Yang-Ming University Hospital, I-Lan 260, Taiwan; E-Mail: fengshulin168@gmail.com; 4 School of Medicine, National Yang-Ming University, Taipei 112, Taiwan; E-Mail: sslo@ymuh.ym.edu.tw; 5 Department of Medical Research and Education, National Yang-Ming University Hospital, I-Lan 260, Taiwan; 6 Institute of Hospital and Health Care Administration, National Yang-Ming University, Taipei 112, Taiwan; 7 Department of Ophthalmology, Chang Gung Memorial Hospital, Taoyuan 333, Taiwan; E-Mail: laurajl@gmail.com; 8 College of Medicine, Chang Gung University, Taoyuan 333, Taiwan; 9 Institute of Pharmacology, National Yang-Ming University, Taipei 112, Taiwan

**Keywords:** statins, hyperlipidemia, lumbar spine injury, national health insurance

## Abstract

The disabling pathophysiologic effects of lipid and neuroprotective effects of statins have recently been demonstrated for acute spinal cord injuries in animal models. This large scale population-based study aimed to investigate the effect hyperlipidemia and the use of statins in patients with lumbar spine injury. The National Health Insurance Research Database of Taiwan was used to identify patients with lumbar spine injury. A total of 2844 patients were grouped into three: no hyperlipidemia, hyperlipidemia using low-dose of statins (≤90 of the defined daily dosage (DDD)), and severe hyperlipidemia using high-dose of statins (>90 DDD). A Cox multiple regression model was used to compare the incidence rates of disability among the three groups. The results showed that patients with hyperlipidemia appeared a higher risk of permanent disability (adjusted HR = 1.38, *p* = 0.28). In subgroup analysis, patients with severe hyperlipidemia had a higher risk of disability (adjusted HR = 3.1, *p* < 0.004), whereas hyperlipidemia using low-dose statins had a similar risk of permanently disability (adjusted HR = 0.83, *p* = 0.661). Hyperlipidemia adversely affected the neurological outcomes of lumbar spinal injury. Statins may have the potential to reverse this higher risk of disability. However, this beneficiary effect of statins only existed in patients using a lower dose (≤90 DDD).

## 1. Introduction

Spinal cord injuries (SCI) can be followed by a process of progressive neurodegeneration with permanent disability. The extent of functional outcome of SCI had been linked to neurotoxicity, oxidative stress and cellular inflammation resulted from peroxidation of lipid [1,2,3]. Statins, inhibitors of 3-hydroxy-3-methyl-glutaryl-CoA reductase (HMG-CoA reductase), are commonly prescribed worldwide for patients with hyperlipidemia. The lipid-lowering efficacy and safety of statins have been established in many clinical trials to reduce the risk of major vascular events such as coronary heart disease, stroke and mortality [4,5,6,7,8]. In recent decades, there have been studies demonstrating the anti-inflammatory and neuroprotective effects of statins [9,10,11]. Lipid-lowering medication is also effective in the treatment or prevention of many diseases of the nervous system, including cerebrovascular disease, Parkinson’s disease, Alzheimer’s disease, multiple sclerosis, and traumatic brain injury [12,13,14,15,16,17]. Animal studies have demonstrated that Simvastatin can ameliorate cauda equine syndrome and attenuate hindlimb dysfunction in spinal cord injury [18,19]. Atorvastatin was also demonstrated to prevent early apoptosis and promote locomotion recovery after spinal cord injury (SCI) in rats [20]. However, the actual role of hyperlipidemia and the effect of statins are not clear in the clinical setting of spinal cord injury.

This study aimed to investigate the effect of hyperlipidemia in the neurological outcomes among patients with lumbar spinal trauma and the potential neuroprotection of statins. There has not been any clinical trial of statins in SCI; therefore, whether pre-existing hyperlipidemia affects recovery of the neurological function in these patients remains uncertain. The authors used the National Health Insurance Research Database (NHIRD) of Taiwan, a database covering the entire Taiwanese population of 23 million for more than 15 years since 1997. This study took advantage of the universal coverage of National Health Insurance (NHI) of Taiwan for a comprehensive follow up of every patient enrolled. The safety of various kinds of statins has been established and they are the standard of care for hyperlipidemia in the NHI. Therefore, the retrospective cohort study can simulate a clinical trial to evaluate the effect of hyperlipidemia and the efficacy of statins in patients with lumbar spine fracture.

## 2. Material and Methods

### 2.1. Ethics

The study was approved by the institutional review board of Taipei Veteran’s General Hospital, Taiwan (IRB# 2012-10-008BC). The database, NHIRD, used in this study is a large computerized de-identified databases derived from the Bureau of National Health Insurance, Taiwan (BNHI) and maintained by the National Health Research Institutes, Taiwan (http://nhird.nhri.org.tw/en/index.htm). The database is publicly available to scientists in Taiwan for research purposes [21].

### 2.2. Enrollment of Patients with Lumbar Trauma

This study used a large dataset from the NHIRD, which was composed of all claims from Taiwan’s NHI program since 1996. To protect privacy, the dataset used in this study is de-identified by the Bureau of National Health Insurance, Taiwan (BNHI) and then encrypted by the National Health Research Institutes, Taiwan, prior to its being used for research purposes (http://nhird.nhri.org.tw/en/index.htm). All diagnoses were recorded by the International Classification of Disease, 9th Version (ICD-9) in the NHIRD. Between 1998 and 2010, subjects aged between 40 and 80 years with the diagnostic codes of 806.4 and 806.5 (fractures of the lumbar vertebral column with SCI) were identified from the study cohort. Those subjects with previous SCI, previous disability, or who had died within one year after SCI were excluded from the current study. Because the study focused on the use of statins for hyperlipidemia, subjects who previously used other anti-hyperlipidemia medication (other classes of lipid-lowering agents such as fibrates, niacin, bile acid sequestrants, or ezetimibe, *etc.*) were also excluded.

### 2.3. Hyperlipidemia and Use of Statins

For every patient enrolled for lumbar spine fracture, as mentioned above, the history of medication use was traced back for one year prior to the incidence of lumbar injury. The use of any kind of statins for hyperlipidemia were identified and analyzed.

There are various kinds of statins, including Simvastatin, Lovastatin, Pravastatin, Fluvastatin, Atorvastatin, Cerivastatin, Rosuvastatin and Pitavastatin, and the single-payer health insurance program of Taiwan, NHI, covered all the costs. To standardize the consumption of various kinds of statins, the authors used a defined daily dose (DDD) that was defined by the World Health Organization (WHO). By the WHO’s definition, the DDD is the assumed average maintenance dose per day for a drug used for its main indication in adults.

Patients who had been using any kinds of statins within one year prior to the lumbar spine injury were identified as those with pre-existing hyperlipidemia. Thus, all patients were grouped into three according to hyperlipidemia (yes or no) and the dosage (high or low dose) of statins: no hyperlipidemia, low-dose, and high-dose groups. Patients who did not use any statins were assigned to the no hyperlipidemia group. Patients who had received statins equal to or less than 90 DDDs were assigned to the low-dose group. Patients who had received statins for more than 90 DDDs were assigned to the high-dose group.

### 2.4. Neurological Outcomes and Other Medical Comorbidities

Lumbar vertebral fracture initially presented with SCI can cause reversible neurological deficits, permanent hemiplegia, or permanent cauda equine syndrome. The study applied the registry of patients in the NHIRD with catastrophic illnesses to identify patients with SCI, causing moderate and severe disabilities permanently. All newly diagnosed lumbar spinal fracture comorbidities with SCI during the follow-up were identified. Therefore, patients with severe neurological deficits such as paraplegia, cauda equine syndrome or incontinence were analyzed at the end of the study period. The incidence of permanently disabled SCI were compared among the groups.

Medical comorbidities, including diabetes mellitus (ICD-9 code, 250.x), hypertension (401-5.x), arrhythmia (426-7.x), and cardiovascular disease (410-4.x), were designated as covariates and adjusted for comparison. These medical comorbidities were determined by the presence of either diagnostic codes in outpatient records or discharge codes in hospitalization records six months before the dates of enrollment to the date of outcome event or the end of follow-up.

### 2.5. Statistical Analysis

All of the data were calculated using the STATA software for descriptive statistics and contingency tables. The Kaplan-Meier method and log-rank test were used to estimate and compare the incidence rates of disabling SCI of the three groups. A Cox multiple regression model was also fitted for comparison. The hazard ratio was adjusted for age, sex, and underlying medical diseases (diabetes, hypertension, arrhythmia, and cardiovascular disease). A probability value of 0.05 was considered statistically significant.

## 3. Results

**Figure 1:**
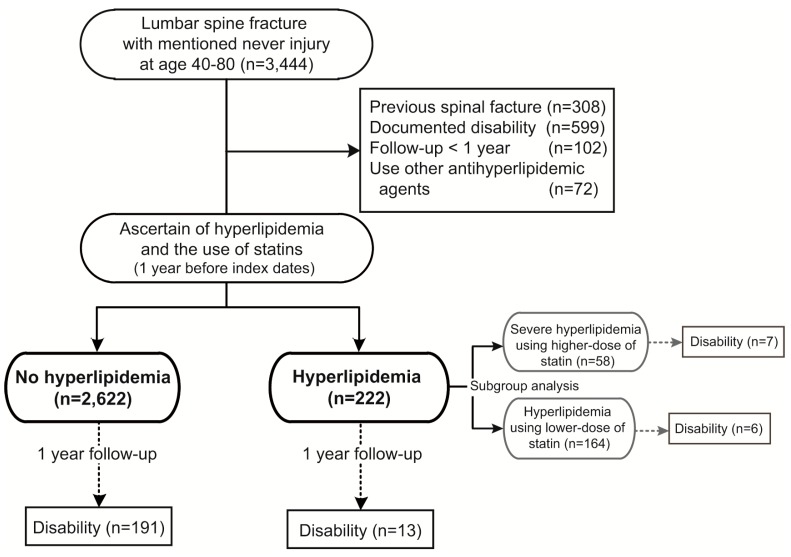
Flow of data processing (1998–2010, Lumbar spine fracture with nerve injury).

A total of 3444 patients with lumbar spine fracture causing SCI were identified from the national cohort. After exclusion of patients with previous spinal fracture, disability, or who were followed up for less than one year, and those who had used any kind of anti-hyperlipidemic agents other than statins, a total of 2844 patients were followed-up. There were 2622 patients who had no hyperlipidemia and used no statins (no hyperlipidemia group). Among the 222 patients who had hyperlipidemia and had taken statins, 164 were using low dose (*i.e.*, equal to or less than 90 DDDs), and the other 58 patients were using high dose (*i.e.*, more than 90 DDDs) of statins implied severe hyperlipidemia (Figure 1).

Gender, age and medical comorbidities were compared among the three groups. Significant differences were demonstrated in gender distribution, age at injury, and underlying medical diseases (all *p* < 0.001) (Table 1).

**Table 1:** Demographic features of patients with lumbar spine injury (*n* = 2844, 1998–2010, Taiwan).

	No Hyperlipidemia		Hyperlipidemia Using Low Dose Statins		Severe Hyperlipidemia Using High Dose Statins		
Demographic features	*n* = 2622	(%)	*n* = 164	(%)	*n* = 58	(%)	*p*-value
Gender	<0.001						
Female	1338	(51.0)	113	(68.9)	41	(70.7)	
Male	1284	(49.0)	51	(31.1)	17	(29.3)	
Age at index date (mean ± SD)	61.01	±11.9	64.37	±10.2	67.35	±9.14	<0.001
Underlying disease							
Diabetes	1113	(42.4)	117	(71.3)	45	(77.6)	<0.001
Hypertension	1773	(67.6)	149	(90.9)	54	(93.1)	<0.001
Arrhythmia	802	(30.6)	72	(43.9)	18	(31.0)	<0.001
Cardiovascular disease	1220	(46.5)	116	(70.7)	35	(60.3)	<0.001
Outcome							
Disabled spinal cord injury	191	(7.3)	6	(3.7)	7	(12.1)	<0.001

### 3.1. Hyperlipidemia Caused more Permanent Disability

**Figure 2:**
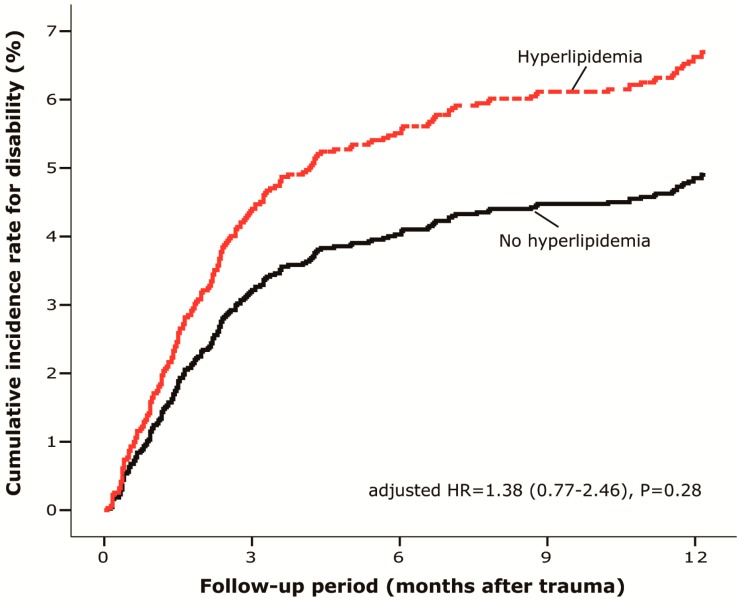
Cumulative incidence rate of permanent disability after lumbar SCI: Hyperlipidemia *vs.* normal subjects. (*n* = 2844, 1998–2010, Taiwan).

In the cohort, there were 2844 patients who had SCI caused by lumbar spine fracture. Of these, 204 had permanent disability at the end of follow-up. The patients with hyperlipidemia appeared with a higher rate of disability during the follow-up (adjusted hazard ratio = 1.38, *p* = 0.28). The cumulative incidence rate for this permanent disability looked lower in patients without hyperlipidemia although no statistical significance (Figure 2).

### 3.2. Lower Dose Statins Might Improve Neurological Outcomes

The study sought to investigate whether the worse neurological outcome (permanently disability) in patients with hyperlipidemia, compared to patients without, could be reversed by the use of a lipid-lowering agent, *i.e.*, statins. Therefore, the 222 hyperlipidemia patients were divided by their frequency and duration of using statins.

**Figure 3:**
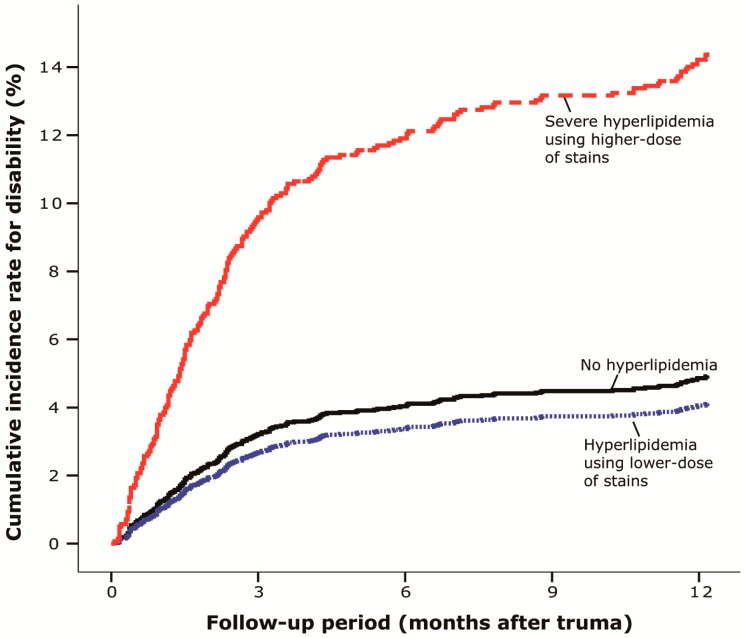
Cumulative incidence rate of permanent disability after lumbar SCI: Subgroup analysis among the normal, hyperlipidemia and severe hyperlipidemia groups. (*n* = 2844, 1998–2010, Taiwan).

The groups using lower and higher dose of statins had very different neurological outcomes. The cumulative incidence rate of permanent disability in the group of patients with hyperlipidemia using low-dose of statins was similar to the no hyperlipidemia group. However, patients with hyperlipidemia using high-dose statins had a significantly higher cumulative incidence rate of permanent disability than the other two groups (Figure 3).

The incidence rate of permanent disability in the hyperlipidemia using lower-dose group appeared lower than the no hyperlipidemia group (3.77 *vs.* 7.69 per 100 person-years, *p* = 0.962). However, the incidence rate of permanent disability in the hyperlipidemia using higher-dose group was significantly higher than the no hyperlipidemia group (13.33 *vs.* 7.69 per 100 person-years, *p* < 0.001). After adjustment for age, sex and underlying disease, patients in the hyperlipidemia using higher-dose statins group were more likely to have permanent disability (adjusted HR = 3.01, *p* = 0.004) than the no hyperlipidemia group. Furthermore, patients in the hyperlipidemia using lower-dose statins group were insignificantly different in the likelihood of disability than the no hyperlipidemia group (adjusted HR = 0.83, *p* = 0.661) (Table 2).

**Table 2:** Incidence rate for permanent disability after lumbar spine injury by hyperlipidemia and use of statins (*n* = 2844, 1998–2010, Taiwan).

Cumulative Usage of Statins in 1 year before Index Date	Incidence Rate for Disability (95% C.I.) (100 person-year)		Crude Incidence Rate Ratio (95% C.I.)			Adjusted Hazard Ratio (95% C.I.)*			*p* value	Sig
No hyperlipidemia ( *n* = 2622)	7.69	(6.67–8.86)		-ref-			-ref			
Hyperlipidemia using lower dose of statins ( *n* = 164)	3.77	(1.69–8.39)	0.49	(0.18–1.09)		0.83	(0.36–1.90)		0.661	
Hyperlipidemia using higher dose of statins ( *n* = 58)	13.33	(6.36–27.97)	1.73	(0.69–3.64)		3.01	(1.43–6.62)		0.004	**

* A Cox multiple regression model was fitted. The hazard ratio was adjusted for age, sex, underlying diseases (diabetes, hypertension, arrhythmia, cardiovascular disease).

## 4. Discussion

This is the first study of humans demonstrating the adverse effect of hyperlipidemia and the possible benefit of statins on the neurological outcomes in patients with lumbar SCI. The present study first compared 2622 patients without hyperlipidemia to 222 patients with hyperlipidemia those subdivided into 164 patients who had used lower-dose of statins (≤90 DDDs) and 58 patients who had hyperlipidemia and used a high-dose of statins (>90 DDDs) in a subgroup analysis. The incidence rate of permanent disability was significantly higher (more than twice as high) in patients with hyperlipidemia than those without (Figure 2). In the subgroup analysis, the lower-dose statin group had a very similar accumulative incidence rate of permanent disability to the no hyperlipidemia (*i.e.*, who had no statins) group. (Figure 3) On the other hand, those patients who had hyperlipidemia using higher-dose of statins were approximately 3 times more likely to develop permanently disability than the no hyperlipidemia group. (Table 2) It has been proposed to avoid hyperlipidemia in SCI by dietary control for improvement in neurological function [22]. However, hyperlipidemia has not been correlated with worse neurological prognosis for SCI in humans. Furthermore, there has been scant data on the effects of statins on SCI among humans, although statins have been demonstrated to be effective in traumatic brain injury [15]. Since there have been only animal studies addressing the efficacy of statins in treating cauda equine syndrome [19], this current study was specifically tailored to evaluate the potential effect of statins for humans with lumbar spinal injury.

The pathophysiology of SCI is comprised of a primary mechanical insult and a cascade of secondary reactions that cause inflammation and neuronal death. The secondary injury involves a series of molecular responses, including neurotoxicity, oxidative stress and cellular inflammation. Studies have demonstrated that the extent and duration of the secondary injury are critical in the determination of the functional outcome after primary injury [1]. Emerging data on the anti-inflammation and neuroprotective effects of statins, HMG Co-A reductase inhibitors, have brought to attention the class of agents in the management of SCI [1,19,20,23,24,25,26]. The pleiotropic effects of statins were demonstrated in several animal studies during the past decade. Pannu *et al.* demonstrated in 2005 and 2007 that rats treated with atorvastatin had higher locomotor ratings after SCI than untreated rats [1,26]. In 2009, Dery *et al.* conducted an experiment showing that atorvastatin promoted locomotor recovery and prevented early cellular apoptosis after SCI [20]. A study of simvastatin in rats conducted by Shunmugavel *et al.* in 2010 revealed amelioration of bladder and renal dysfunction after SCI [19]. In 2012, Han *et al.* demonstrated that simvastatin in rats promoted functional recovery after SCI by migrating bone marrow stromal cells [24].

The statins seemed to intervene in the secondary injury via various reactions, most of which were associated with the blood-spinal cord barrier (BSCB) [1]. BSCB was considered central to secondary neuronal injury. Pannu *et al.* demonstrated that atorvastatin prevented BSCB breakdown by attenuation of matrix metalloproteinase (MMP, especially MMP9), which was related to neutrophil/macrophage and inflammatory cytokine accumulation after neural injury [1,25,26]. Atorvastatin also revealed the ability to reduce reactive astrocytic activity and attenuated reactive astrogliosis that was associated with the down-regulation of the inducible nitric oxide synthase (iNOS) pathway, and inhibition of cellular infiltration of TNF-α and interleukin-1β [1,23,26]. In 2013, simvastatin was demonstrated to express similar actions in another study on rats [1,19]. Another crucial pathway in post-SCI degeneration is injury-induced GTPase Rho activation [1,23]. Statins indirectly deplete the supply of isoprenoids by inhibiting cholesterol synthesis, and such reduction of Rho activity was shown to prevent endothelial dysfunction and cellular apoptosis. Such a pathway was also involved in the reduction of MMP9 mRNA expression [1]. Besides the central nervous system, the attenuation of neuronal damage by statins was also observed in peripheral nerves via similar mechanisms. In 2010, Pan *et al.* simulated a crush injury of sciatic nerves in rats and treated with atorvastatin [25]. The study suggested that atorvastatin reduced BSCB permeability by decreasing MMP-2/MM-9 activity, promoted nerve regeneration and modulated intracellular signaling, which was demonstrated by reduced injury-induced phosphorylation of ERK, Akt and STAT1. In summation, these reactions suggested that statins expressed the ability of attenuation of injury-induced tissue necrosis, demyelination, apoptosis, blocking cellular infiltration, reduction of secondary inflammation, and ameliorating Wallerian degeneration and reactive gliosis after neuronal injury.

The protection effect of statins was demonstrated in the present study only for the group of patients with hyperlipidemia using lower-dose (*i.e.*, equal to or less than 90 DDDs) of statins. Higher-dose of statins was associated with worse neurological outcomes after lumbar spinal injury in patients with hyperlipidemia. This paradoxical phenomenon could be attributed to the severity of hyperlipidemia, partial response to the statins, or poor compliance to the prescriptions. For example, patients who used a higher DDD very likely had higher serum cholesterol levels or more medical comorbidities that could cause more disability. Statins, despite their potential neuroprotection, might not be enough to overcome the adverse effects of severe hyperlipidemia and other medical comorbidities. Different doses of statins, ranging from 1 mg/kg to 20 mg/kg, have been tested in previous studies using animal models [1,20,23,24,26]. Due to the different sensitivity among species and adverse effects at high doses, scrutinized dose design and larger investigations should be conducted to define an optimal therapeutic level in humans. The criteria of 90 DDD in the present study was the standard dose of statins in a single-course of treatment for hypercholesterolemia regulated by the NHI program in Taiwan.

There are several limitations to the present study. First, the use of statins was defined by prescription claims. Prescription dosage of statins does not necessarily equal the actual consumption dosage. Second, the patients of hyperlipidemia in this cohort study were identified according to the prescription of statins rather than the actual serum lipid concentration. It is possible that hyperlipidemia was under-diagnosed in this cohort if the patient did not seek medical service prior to the indexed date (*i.e.*, the event of lumbar spine injury). Third, the detailed records of neurological deficits caused by lumbar spine injury were not available in NHIRD. However, theoretically there were no correlations between hyperlipidemia or the use of statins and the severity of lumbar spine injury. Therefore, patients in each of the three groups (no hyperlipidemia, hyperlipidemia using lower-dose, and hyperlipidemia using higher-dose of statins) should have a similar distribution of severity of injury. Although details were lacking, the comparison of the incidence rates of permanent disability between the groups was thus valid. The event of lumbar spine injury could be regarded as a date for randomization in the cohort. Little selection bias existed between the three groups because the randomization was based on the history of drug prescriptions, which was prudently recorded in NHIRD and under constant internal monitoring. It is impossible to conduct a prospective randomized clinical study to allocate patients by injury. This cohort study therefore provided valuable data on the association between statins and neurological outcomes after lumbar spine injury.

## 5. Conclusions

In this cohort of patients with lumbar spinal injury, hyperlipidemia adversely affected the neurological outcomes and caused more permanent disability. Statins may have the potential to reverse this higher risk of disability. However, this beneficiary effect of statins only existed in patients using a lower dose (≤90 DDD). Further investigations for using statins in SCI patients are required to corroborate their neuroprotective effect.

## References

[1] Pannu R., Christie D.K., Barbosa E., Singh I., Singh A.K. (2007). Post-trauma Lipitor treatment prevents endothelial dysfunction, facilitates neuroprotection, and promotes locomotor recovery following spinal cord injury. J. Neurochem..

[2] Yip P.K., Malaspina A. (2012). Spinal cord trauma and the molecular point of no return. Mol. Neurodegener..

[3] Jia Z., Zhu H., Li J., Wang X., Misra H., Li Y. (2012). Oxidative stress in spinal cord injury and antioxidant-based intervention. Spinal Cord.

[4] Baigent C., Keech A., Kearney P.M., Blackwell L., Buck G., Pollicino C., Kirby A., Sourjina T., Peto R., Collins R. (2005). Efficacy and safety of cholesterol-lowering treatment: Prospective meta-analysis of data from 90,056 participants in 14 randomised trials of statins. Lancet.

[5] Gould A.L., Rossouw J.E., Santanello N.C., Heyse J.F., Furberg C.D. (1998). Cholesterol reduction yields clinical benefit: Impact of statin trials. Circulation.

[6] LaRosa J.C., He J., Vupputuri S. (1999). Effect of statins on risk of coronary disease: A meta-analysis of randomized controlled trials. JAMA.

[7] Schwartz G.G., Olsson A.G., Ezekowitz M.D., Ganz P., Oliver M.F., Waters D., Zeiher A., Chaitman B.R., Leslie S., Stern T. (2001). Effects of atorvastatin on early recurrent ischemic events in acute coronary syndromes: The MIRACL study: A randomized controlled trial. JAMA.

[8] Shepherd J., Blauw G.J., Murphy M.B., Bollen E.L., Buckley B.M., Cobbe S.M., Ford I., Gaw A., Hyland M., Jukema J.W. (2002). Pravastatin in elderly individuals at risk of vascular disease (PROSPER): A randomised controlled trial. Lancet.

[9] Duval D. (2000). Effects of statins on ischemic stroke: Neuroprotection and/or triggering of apoptotic damage?. Stroke.

[10] Kumar A., Sharma N., Mishra J., Kalonia H. (2013). Synergistical neuroprotection of rofecoxib and statins against malonic acid induced Huntington’s disease like symptoms and related cognitive dysfunction in rats. Eur. J. Pharmacol..

[11] Wood W.G., Eckert G.P., Igbavboa U., Muller W.E. (2010). Statins and neuroprotection: A prescription to move the field forward. Ann. N. Y. Acad. Sci..

[12] Eckert G.P., Wood W.G., Muller W.E. (2005). Statins: Drugs for Alzheimer’s disease?. J. Neural Transm..

[13] Nassief A., Marsh J.D. (2008). Statin therapy for stroke prevention. Stroke.

[14] Neuhaus O., Hartung H.P. (2007). Evaluation of atorvastatin and simvastatin for treatment of multiple sclerosis. Expert Rev. Neurother..

[15] Tapia-Perez J., Sanchez-Aguilar M., Torres-Corzo J.G., Gordillo-Moscoso A., Martinez-Perez P., Madeville P., de la Cruz-Mendoza E., Chalita-Williams J. (2008). Effect of rosuvastatin on amnesia and disorientation after traumatic brain injury (NCT003229758). J. Neurotrauma.

[16] Undela K., Gudala K., Malla S., Bansal D. (2013). Statin use and risk of Parkinson’s disease: A meta-analysis of observational studies. J. Neurol..

[17] Wahner A.D., Bronstein J.M., Bordelon Y.M., Ritz B. (2008). Statin use and the risk of Parkinson disease. Neurology.

[18] Saito T., Tsuchida M., Umehara S., Kohno T., Yamamoto H., Hayashi J. (2011). Reduction of spinal cord ischemia/reperfusion injury with simvastatin in rats. Anesth. Analg..

[19] Shunmugavel A., Martin M.M., Khan M., Copay A.G., Subach B.R., Schuler T.C., Singh I. (2013). Simvastatin ameliorates cauda equina compression injury in a rat model of lumbar spinal stenosis. J. Neuroimmune Pharmacol..

[20] Dery M.A., Rousseau G., Benderdour M., Beaumont E. (2009). Atorvastatin prevents early apoptosis after thoracic spinal cord contusion injury and promotes locomotion recovery. Neurosci. Lett..

[21] Chen Y.C., Yeh H.Y., Wu J.C., Haschler I., Chen T.J., Wetter T. (2011). Taiwan’s National Health Insurance Research Database: Administrative health care database as study object in bibliometrics. Scientometrics.

[22] Plunet W.T., Streijger F., Lam C.K., Lee J.H., Liu J., Tetzlaff W. (2008). Dietary restriction started after spinal cord injury improves functional recovery. Exp. Neurol..

[23] Die J., Wang K., Fan L., Jiang Y., Shi Z. (2010). Rosuvastatin preconditioning provides neuroprotection against spinal cord ischemia in rats through modulating nitric oxide synthase expressions. Brain Res..

[24] Han X., Yang N., Cui Y., Xu Y., Dang G., Song C. (2012). Simvastatin mobilizes bone marrow stromal cells migrating to injured areas and promotes functional recovery after spinal cord injury in the rat. Neurosci. Lett..

[25] Pan H.C., Yang D.Y., Ou Y.C., Ho S.P., Cheng F.C., Chen C.J. (2010). Neuroprotective effect of atorvastatin in an experimental model of nerve crush injury. Neurosurgery.

[26] Pannu R., Barbosa E., Singh A.K., Singh I. (2005). Attenuation of acute inflammatory response by atorvastatin after spinal cord injury in rats. J. Neurosci. Res..

